# Ontology-based approaches for multi-destination tourism planning: a systematic literature review

**DOI:** 10.3389/frai.2026.1881434

**Published:** 2026-07-20

**Authors:** Ummi Maisarah Izani, Nur Syadhila Che Lah

**Affiliations:** Department of Computing, Universiti Teknologi PETRONAS (UTP), Perak, Malaysia

**Keywords:** knowledge graph, knowledge representation, ontology, smart tourism, systematic literature review

## Abstract

Tourism planning is becoming increasingly complex as travel behavior shifts from single-destination visits to multi-destination itineraries. However, many tourism information systems still rely on point-of-interest data and recommendation algorithms that lack the semantic structures needed to represent relationships between destinations. This limitation is important in smart tourism environments that require interoperable, data-integrated systems to support meaningful travel planning. This study conducts a systematic literature review of ontology and knowledge graph-based approaches for tourism planning to assess their ability to support itinerary modelling. Using PRISMA guidelines, relevant studies were retrieved from Scopus, ScienceDirect and IEEE Xplore, with 21 articles meeting all eligibility criteria. The review identifies six themes: ontology and semantic modelling, knowledge graph construction and enrichment, tourism recommendation and personalization, semantic retrieval and question answering, route planning and decision support, and itinerary-level and multi-destination representation. The analysis shows that ontology and knowledge graphs are widely used to organize tourism knowledge and enhance recommendation systems. However, most studies remain focused on entity-level modelling, while explicit semantic modelling of itinerary-level constructs remains limited. This gap highlights the need for ontology frameworks that can formally represent travel sequences and inter-destination dependencies to better support smart-tourism planning.

## 1 Introduction

Tourism has undergone major transformation in recent decades as travel behavior shifts from single-destination visits to complex multi-destination itineraries. This form of travel involves a sequence of destinations within one trip, driven by motivations to maximize experience, optimize cost and time, and explore connected attractions within a region (Lew and McKercher, 2006). As mobility becomes more interconnected, planning systems must represent relationships between destinations rather than treating each location as an isolated point of interest.

Multi-destination travel introduces considerable complexity to tourism planning. Unlike traditional models that focus on individual destinations, multi-destination tourism requires consideration of spatial relationships, travel sequences, temporal constraints, transportation availability, and traveler preferences. However, many existing tourism information systems remain data-driven or platform-centric. They rely heavily on recommendation algorithms and isolated point-of-interest datasets, lacking structured mechanisms to represent semantic relationships between tourism entities and travel sequences (Gretzel et al., 2015). In destinations where diverse attractions and stakeholders coexist, the need for integrated and semantically structured systems becomes critical. Smart tourism initiatives depend on data-driven platforms that support mobility, enhance visitor experience, and improve resource management (Gretzel et al., 2015; Xiang et al., 2015). In this context, knowledge representation plays a key role in enabling interoperability and intelligent services (Gómez-Pérez, 1999; Chessa et al., 2023).

Ontology-based approaches have therefore been widely adopted to represent complex knowledge domains. Ontology is defined as an explicit specification of a shared conceptualization that formally describes domain concepts, relationships, and constraints in machine-interpretable form (Gruber, 1993). Using semantic frameworks such as the Resource Description Framework (RDF) and the Web Ontology Language (OWL), ontology models integrate heterogeneous data sources while maintaining semantic consistency (Noy and McGuinness, 2001). They enhance semantic clarity, facilitate knowledge reuse, and promote interoperability across distributed systems (Feilmayr and Wöß, 2016). Beyond its technical function, ontology theory provides a formal epistemological basis for representing and reasoning about domain knowledge. Ontologies incorporate axioms, constraints, and inference rules that enable machines to interpret meaning across disparate systems (Gruber, 1993; Gómez-Pérez, 1999). Within knowledge-representation theory, ontology acts as a semantic bridge that supports consistent meaning exchange and structural interoperability between diverse data sources (Noy and McGuinness, 2001; Uschold and Gruninger, 1996; Poveda-Villalón et al., 2022). This theoretical grounding positions ontology not only as an engineering artefact but as a formal representation of shared conceptual understanding in smart tourism ecosystems.

Within tourism, ontology and knowledge graph approaches have been applied in destination knowledge modelling, recommender systems, cultural heritage management, and intelligent tourism platforms. In the Malaysian tourism context, Izani and Che Lah (2025) proposed an ontology-driven tourism insight platform that integrates tourism-related knowledge into semantic entities and relationships to support tourism information access and recommendation. However, most work focuses on individual tourism entities such as destinations, attractions, or services, while multi-destination structures, including itinerary sequences, inter-destination dependencies, and travel transitions, remain weakly modelled. Despite the broad adoption of ontology and knowledge-graph techniques, research remains fragmented across semantic web, recommender system, and data integration contexts. This fragmentation limits a comprehensive understanding of how ontology-based approaches support complex travel planning scenarios.

A systematic literature review (SLR) was selected to examine how ontology and knowledge graph-based approaches have been applied in tourism systems. Ontology-based tourism research is dispersed across multiple streams, including semantic web technologies, knowledge graphs, intelligent tourism systems, and recommendation platforms. Many studies emphasize application-level performance while providing limited discussion of ontology engineering processes, modelling strategies, or validation approaches. Existing reviews have discussed broader areas such as recommender systems and smart tourism ecosystems, but there remains a need to examine how semantic tourism models represent travel relationships such as itinerary structures and inter-destination dependencies (Petrosino et al., 2001; Mallett et al., 2012).

Although tourism technology research has examined recommender systems, smart tourism platforms, and knowledge-based applications, less attention has been given to how ontology and knowledge graph-based tourism systems represent itinerary-level semantics. The present review addresses this gap by analysing whether existing tourism semantic systems remain entity-centred or have progressed toward modelling multi-destination travel structures.

**Figure 1:**
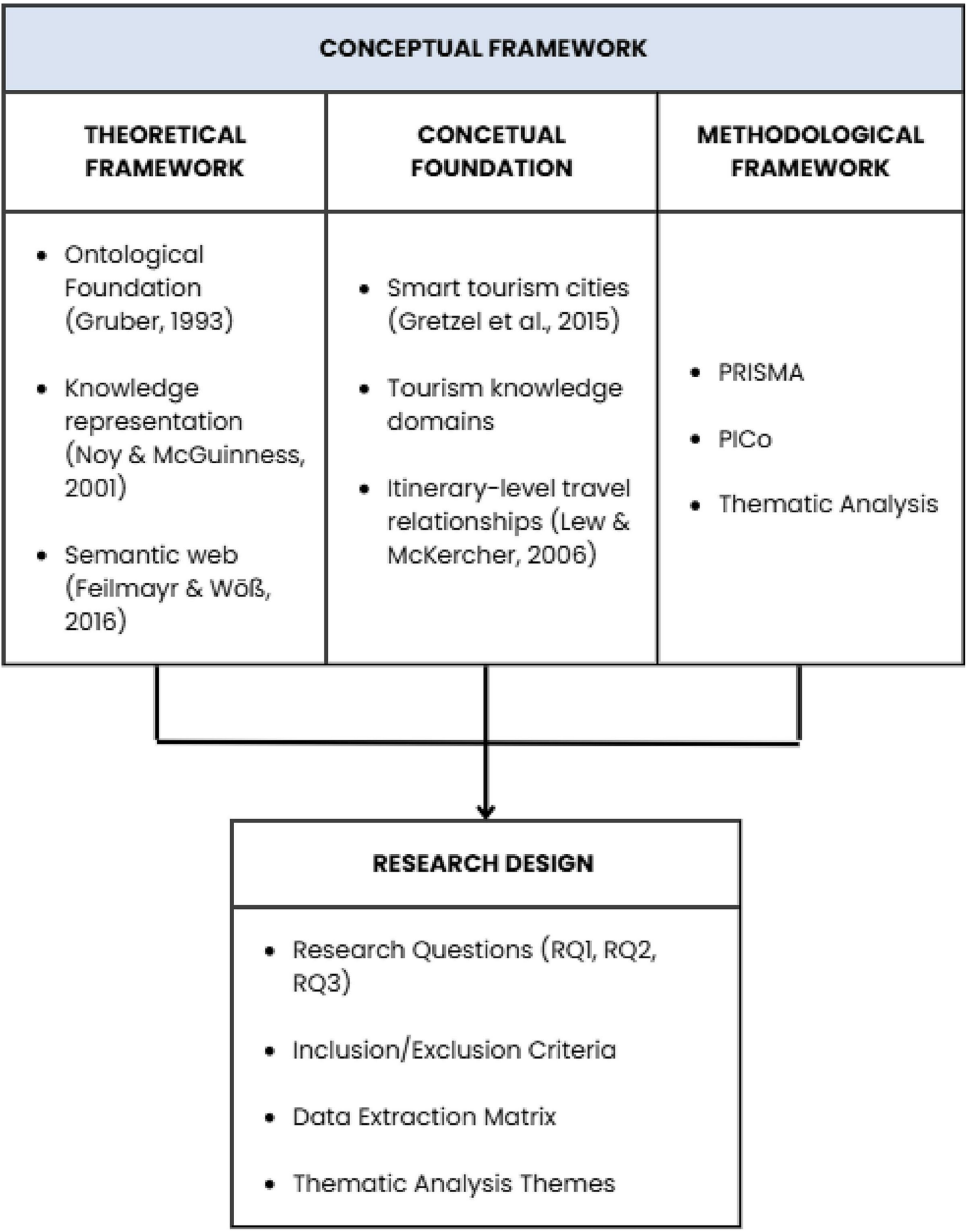
Conceptual framework integrating ontological foundation, multi-destination tourism concept, and systematic review methodology.

To guide the analysis, this study adopts a conceptual framework that integrates three interconnected dimensions: ontology and knowledge graph approaches, multi-destination tourism, and systematic review methodology. Ontology and knowledge graph approaches provide the basis for representing structured knowledge, while multi-destination tourism frames travel behavior as sequences of connected destinations rather than isolated visits (Lew and McKercher, 2006). Methodologically, the study follows PRISMA (Page et al., 2021) and PICo (Joanna Briggs Institute, 2020) to ensure a structured and transparent review process. The conceptual framework adopted in this study is illustrated in Figure 1.

Therefore, the main objective of this study is to systematically review existing literature on ontology and knowledge graph-based approaches applied in tourism systems, with particular emphasis on their capability to support multi-destination tourism planning. Specifically, this review aims to (1) examine the ontology and knowledge graph approaches used in tourism systems, (2) analyze their design, structure and validation practices and (3) identify limitations in the representation of multi-destination travel relationships.

## 2 Materials and methods

### 2.1 PRISMA

This study adopts the Preferred Reporting Items for Systematic Reviews and Meta-Analyses (PRISMA) 2020 framework to guide the reporting of the systematic literature review process (Page et al., 2021). PRISMA was used to structure the identification, screening, eligibility assessment, and inclusion stages of the review. Although originally introduced within medical research, PRISMA has been widely applied across multidisciplinary domains including environmental management, information systems and technology research due to its structured review procedures (Moher et al., 2009; Sierra-Correa and Cantera Kintz, 2015).

PRISMA is appropriate for this study because the reviewed literature is distributed across tourism informatics, semantic web technologies, recommendation systems, smart tourism and knowledge engineering. The framework helped organize the search and selection process across multiple databases and provided a clear structure for reporting how studies were identified and selected.

**Figure 2:**
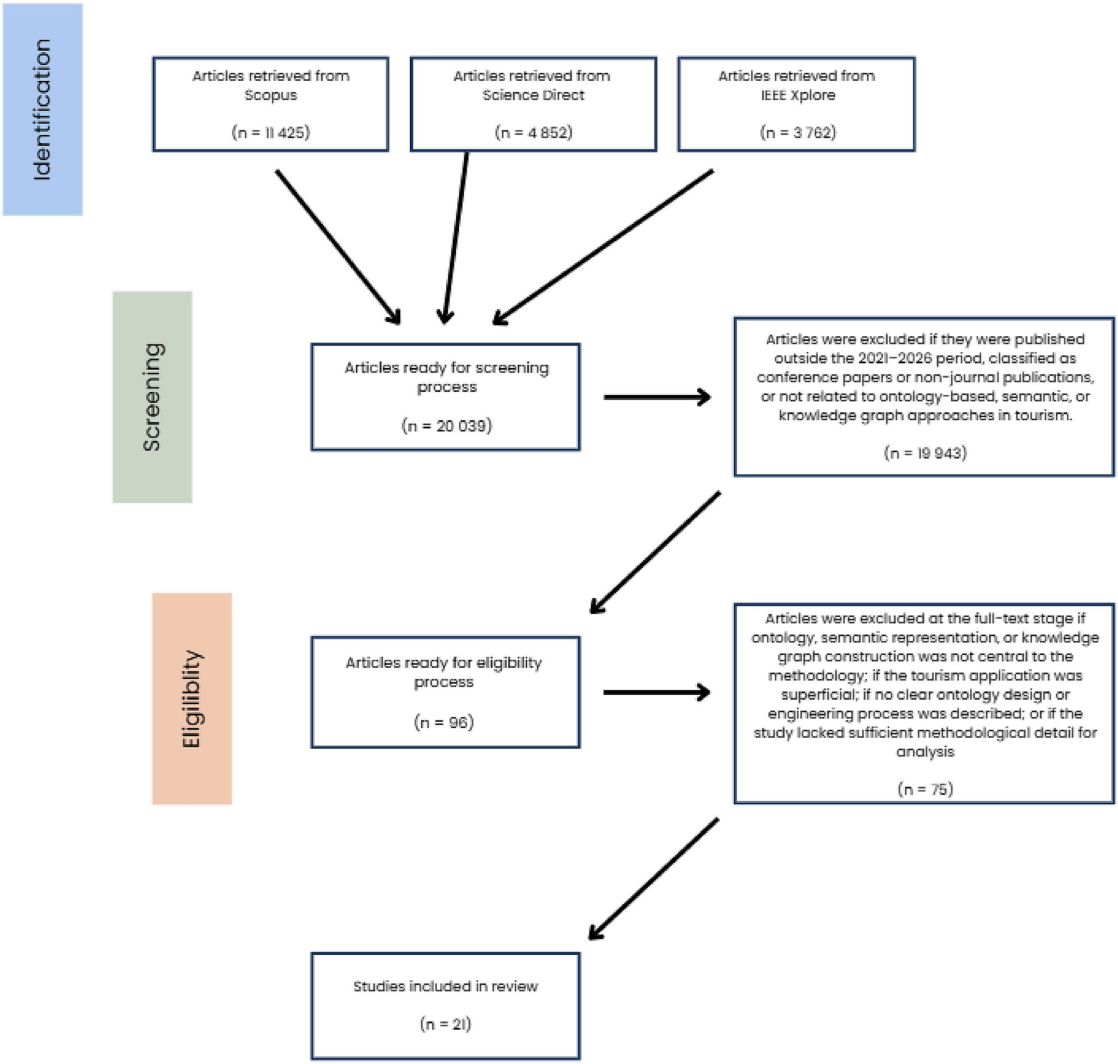
PRISMA flow diagram illustrating the study selection process.

Following PRISMA, the review process consisted of four stages: identification, screening, eligibility assessment and final inclusion. The entire process is summarized in the PRISMA flow diagram presented in Figure 2. Further details on database searches, screening criteria, eligibility assessment, and quality assessment are reported in the following subsections.

### 2.2 Formulation of the research question

The research questions of this study were formulated using the PICo (Population–Interest–Context) framework. PICo is commonly employed in qualitative and conceptual systematic reviews to provide clarity in scope definition and alignment between research objectives and search strategy (Joanna Briggs Institute, 2020). Compared with the PICO framework used in clinical intervention studies, PICo is more suitable for exploratory domains such as ontology engineering, knowledge graph applications and tourism informatics.

In this study, the PICo components were defined as follows:

- *Population (P)*: Tourism systems using ontology or knowledge graph approaches
- *Interest (I)*: Semantic modeling techniques and validation methods
- *Context (Co)*: Tourism planning and multi-destination itinerary semantics.

Guided by the PICo structure, the following research questions (RQ) were developed:

- *RQ1*: What ontology and knowledge graph-based approaches have been applied in tourism planning and related tourism decision-support systems?
- *RQ2*: How are these approaches designed, structured and validated in the reviewed studies?
- *RQ3*: To what extent do existing ontology and knowledge graph-based tourism approaches represent itinerary-level semantics?

This formulation aligns the research questions with the revised screening and data extraction process. Multi-destination itinerary semantics is treated as an analytical focus rather than a mandatory feature of every included study. Therefore, studies on single-destination, attraction-level, accommodation-level, cultural tourism, recommendation, or semantic retrieval systems were retained when they used ontology or knowledge graph approaches and helped assess the extent of itinerary-level representation.

### 2.3 The systematic review process for selecting articles

#### 2.3.1 Identification

The identification stage aimed to retrieve a comprehensive pool of potentially relevant studies through structured database searches. These databases were selected due to their extensive coverage of interdisciplinary publications spanning tourism research, information systems, semantic web technologies and knowledge engineering.

The search was conducted across three major academic databases:

- Scopus
- ScienceDirect
- IEEE Xplore

The database searches were conducted in February 2026. The Scopus search was conducted from 9 to 11 February 2026, followed by IEEE Xplore from 13 to 19 February 2026 and ScienceDirect from 20 to 24 February 2026. Search queries were developed using Boolean operators, keyword combinations and truncation symbols. The search terms were organized into three concept clusters: (1) ontology, knowledge graph, and semantic technologies; (2) tourism and tourism planning systems; and (3) itinerary, route, trip, and travel-sequence representation.

Boolean operators (AND, OR) were applied to connect related terms and ensure logical structuring of queries. Truncation symbols (e.g., ontolog*) were used to capture variations such as ontology, ontologies and ontology based. Additional terms such as “tourism planning,” “travel planning,” “travel sequence,” and “travel route” were included to align the search strategy with the review focus on itinerary-level representation. The search strings were adapted according to the syntax requirements of each database. Table 1 presents the final search strings used in this study.

**Table 1:** Database search strings used in the identification stage.

Database	Search string
Scopus	TITLE-ABS-KEY ((ontolog* OR “ontology-based” OR “ontology driven” OR “semantic web” OR “semantic-based” OR “knowledge graph*” OR “knowledge representation” OR “conceptual modeling” OR “concept modelling”) AND (tourism OR “tourism planning” OR “travel planning” OR “travel sequence” OR “travel route” OR “tourism system*” OR “decision support system*” OR “recommendation system*” OR “recommender system*”))
ScienceDirect	(“ontology” OR “ontology-based” OR “semantic web” OR “knowledge graph”) AND (“tourism” OR “tourism planning” OR “travel planning” OR “travel sequence” OR “travel route” OR “tourism system”)
IEEE Xplore	(“Abstract”:ontolog* OR “Abstract”:"ontology-based” OR “Abstract”:"semantic web” OR “Abstract”:"knowledge graph*”) AND (“Abstract”:tourism OR “Abstract”:"tourism planning” OR “Abstract”:"travel planning” OR “Abstract”:"travel sequence” OR “Abstract”:"travel route” OR “Abstract”:"decision support system*” OR “Abstract”:"recommender system*”)

Search strings were adapted to match the syntax requirements of each database.

The identification stage retrieved 20,039 records, consisting of 11,425 articles from Scopus, 4,852 from ScienceDirect and 3,762 from IEEE Xplore. Retrieved records were exported and organized in Mendeley using folders and tags. Duplicate records were checked and removed before title and abstract screening. However, the exact duplicate count was not separately recorded in the original screening worksheet; this is acknowledged as a limitation of the review audit trail.

#### 2.3.2 Screening

The screening phase applied predefined inclusion and exclusion criteria to refine retrieved records. Screening was conducted in two stages. First, database filters were applied based on publication year, document type, and language. Second, titles and abstracts were manually reviewed against the inclusion and exclusion criteria.

A publication timeline filter retained studies published between 2021 and 2026, ensuring alignment with recent developments in ontology-based tourism research (Alexander, 2020). Only peer-reviewed journal articles were included. Conference papers, book chapters, editorials, theses, and non-peer-reviewed publications were excluded.

Titles and abstracts were examined to determine whether each study involved tourism, travel, hospitality, cultural tourism, destination, attraction, accommodation, or related tourism service contexts. Studies were retained when they applied ontology, knowledge graph, semantic web, RDF, OWL, graph schema, rule-based semantic modelling, semantic retrieval, or semantic recommendation approaches. Screening was conducted by one reviewer using the predefined inclusion and exclusion criteria, and borderline cases were discussed with the supervisor during progress meetings. Where uncertainty occurred, the study was retained for full-text assessment rather than excluded at the title and abstract stage. Cohen’s Kappa was not calculated because independent dual-screening records were not available. This limitation is acknowledged, and consistency was supported through predefined criteria, full-text rechecking, and quality assessment.

Following the screening process, 19,943 records were excluded, leaving 96 articles that met the screening criteria and were retained for full-text assessment. The inclusion and exclusion criteria applied during the screening stage are summarized in Table 2.

**Table 2:** Inclusion and exclusion criteria for article selection.

Criterion	Inclusion	Exclusion
Literature type	Peer-reviewed journal articles (empirical or conceptual with ontology implementation)	Review papers, editorials, book chapters, conference proceedings, theses, non-peer-reviewed publications
Language	English	Non-English publications
Timeline	2021–2026	Published before 2021
Research focus	Studies explicitly applying ontology, semantic modelling, or knowledge graph approaches within tourism	Tourism studies without ontology-based modelling; purely machine learning or statistical recommender systems without semantic representation
Application Domain	Tourism planning, itinerary planning, destination modelling, decision support systems in tourism	Non-tourism ontology applications; tourism marketing without planning or modelling component
Methodological detail	Clear ontology design, structure, relationships, or validation method described	Superficial mention of ontology without engineering description or validation

The criteria were revised to align the review with ontology and knowledge graph-based tourism studies. Studies were not required to explicitly model multi-destination itineraries at the inclusion stage. Instead, itinerary-level representation was assessed during data extraction and thematic analysis.

#### 2.3.3 Eligibility

The eligibility stage involved detailed full-text assessment of the remaining 96 articles to determine their conceptual and methodological alignment with the objectives of this review. Full-text evaluation is essential in systematic reviews because abstracts may not sufficiently reflect the depth of methodological implementation (Kitchenham and Charters, 2007).

During this stage, each article was assessed to determine whether ontology modelling was a central methodological component. Attention was given whether the study applied ontology, knowledge graph, semantic web, OWL, RDF graph schema, rule-based semantic modelling, semantic retrieval, or semantic recommendation approaches within a tourism-related context. The assessment also considered whether the study provided sufficient methodological detail for comparison, such as classes, nodes, relationships, semantic schema, reasoning mechanism, retrieval method, recommendation method, validation, or evaluation.

Studies were excluded if they were not related to tourism, did not include ontology, knowledge graph, or semantic modelling, focused only on machine learning or statistical recommendation without semantic structure, lacked sufficient methodological detail, or did not provide any validation or evaluation. Following full-text evaluation, 75 articles were excluded, leaving 21 articles meeting all eligibility and quality requirements for final synthesis.

Summary of review methodology:

- Timeframe: 2021–2026
- Databases: Scopus, ScienceDirect, IEEE Xplore
- Initial records: 20,039
- After screening: 96 articles
- Final included: 21 studies
- Inclusion criteria: Peer-reviewed journal articles, English, tourism-related context, ontology, knowledge graph, or semantic modelling approach
- Exclusion criteria: Conference papers, non-English, studies without ontology, knowledge graph, or semantic modelling, purely ML/statistical recommendation studies without semantic structure

#### 2.3.4 Quality and relevance assessment

A quality assessment was conducted for the retained studies to evaluate their methodological clarity and relevance to the review questions. A custom scoring framework was used because the reviewed articles covered different types of studies, including ontology development, knowledge graph construction, semantic retrieval, recommendation systems, route planning, and decision-support applications.

Each study was assessed using four criteria: (1) relevance to ontology, knowledge graph, or semantic tourism approaches; (2) clarity of ontology or knowledge graph design, including classes, entities, relationships, schema, or modelling pipeline; (3) evidence of itinerary, route, destination relationship, or multi-destination representation; and (4) contribution to identifying gaps or limitations in tourism semantic modelling. Each criterion was scored as 0 = not reported, 0.5 = partially reported, and 1 = clearly reported. The maximum score was 4. Studies scoring 3.0–4.0 were classified as high relevance, 2.0–2.5 as moderate relevance, and below 2.0 as low relevance. The quality assessment results are summarized in Table 3.

**Table 3:** Quality and relevance assessment.

Study	Study reference	C1	C2	C3	C4	Total	Assessment outcome
S1	Zhang (2026)	0.5	0.5	0	0.5	1.5	Low relevance
S2	Wang et al. (2025)	0.5	1	0	0.5	2.0	Moderate relevance
S3	Jiang and Han (2025)	0.5	1	0	0.5	2.0	Moderate relevance
S4	Xu et al. (2025)	0.5	0.5	0	0.5	1.5	Low relevance
S5	Zuo and Li (2025)	0.5	1	0	0.5	2.0	Moderate relevance
S6	Li (2024)	0.5	0.5	1	1	3.0	High relevance
S7	Fan and Chen (2024)	1	1	0	1	3.0	High relevance
S8	Gao et al. (2024)	1	1	0.5	0.5	3.0	High relevance
S9	Ding (2024)	0	0	1	1	2.0	Moderate relevance
S10	Haridy et al. (2023)	1	1	0	1	3.0	High relevance
S11	Chessa et al. (2023)	1	1	0	1	3.0	High relevance
S12	Ojino et al. (2022)	1	1	0	1	3.0	High relevance
S13	Do et al. (2021)	0.5	0.5	0	1	2.0	Moderate relevance
S14	Pinto et al. (2022)	1	1	0.5	1	3.5	High relevance
S15	Casillo et al. (2025)	1	0.5	1	0.5	3.0	High relevance
S16	Aghaei et al. (2022)	1	0.5	0.5	0.5	2.5	Moderate relevance
S17	Gao et al. (2023)	1	0.5	0.5	0.5	2.5	Moderate relevance
S18	Fan and Wang (2022)	0.5	1	0	0.5	2.0	Moderate relevance
S19	Abbasi-Moud et al. (2022)	1	0.5	0	1	2.5	Moderate relevance
S20	Fang et al. (2021)	1	0.5	0	1	2.5	Moderate relevance
S21	Cadeddu et al. (2024)	0.5	0.5	0	1	2.0	Moderate relevance

C1 = relevance to ontology, knowledge graph, or semantic tourism approaches; C2 = clarity of ontology or knowledge graph design; C3 = evidence of itinerary, route, destination relationship, or multi-destination representation; C4 = contribution to identifying gaps or limitations in tourism semantic modelling. Scores were assigned as 0 = not reported, 0.5 = partially reported, and 1 = clearly reported. Total score: 3.0–4.0 = high relevance; 2.0–2.5 = moderate relevance; below 2.0 = low relevance.

The assessment shows that the studies included varied in their relevance to the review questions. Nine studies were classified as high relevance, providing clear ontology or knowledge graph design and strong contributions to the synthesis. Ten studies were classified as moderate relevance, offering useful supporting evidence for specific application domains (e.g., recommendation, QA, cultural heritage). Two studies (S1 and S4) were classified as low relevance but were retained because they illustrate emerging approaches such as multimodal knowledge graph construction. Notably, only three studies (S6, S8, S9) received partial or full credit for C3, confirming the limited attention to itinerary-level and multi-destination semantic modelling in the current literature.

### 2.4 Data extraction and analysis

Data extraction and analysis were conducted on the 21 retained studies. This review adopted an integrative review approach to synthesize findings from different types of semantic tourism research, including ontology implementations, knowledge graph systems, semantic retrieval models, recommendation systems, and route planning applications. Integrative reviews enable systematic analysis of heterogeneous methodological evidence within a unified framework (Whittemore and Knafl, 2005), making this approach appropriate for ontology-based tourism research, which spans formal semantic modelling to applied system development (Torraco, 2016).

Each selected article was examined multiple times to extract information aligned with the research questions. The extraction focused on study characteristics, semantic approach, tourism domain, modelling structure, evaluation method, and itinerary-level representation. The extracted information was then organized into a structured data extraction matrix to facilitate systematic comparison across studies.

#### 2.4.1 Coding and thematic development

Extracted data were analyzed using deductive thematic analysis. The initial coding categories were developed from the research questions and the data extraction fields, rather than emerging only from the raw data (Braun and Clarke, 2006). The coding focused on the type of semantic approach, tourism application, modelling structure, evaluation method, and level of itinerary representation.

The coding process involved three steps. First, each study was coded based on its main semantic approach, such as ontology engineering, knowledge graph construction, semantic retrieval, recommendation, or route planning. Second, studies were coded according to their tourism application, such as cultural heritage, accommodation, attraction recommendation, hospitality, decision support, or itinerary-related planning. Third, each study was assessed for itinerary-level representation by checking whether it included Trip Class, Itinerary Class, Segment Class, Temporal Relation, and Route Semantic.

For example, a study that developed an ontology with class hierarchies and semantic relationships was coded under ontology engineering. A study that constructed linked tourism entities using graph-based data structures was coded under knowledge graph construction. A study that generated routes or recommendations without explicitly modelling Trip or Segment classes was coded as route or recommendation-oriented, but not as explicit itinerary-level modelling.

Based on this process, six themes were used to organize the findings: ontology and semantic modelling, knowledge graph construction and enrichment, tourism recommendation and personalization, semantic retrieval and question answering, route planning and decision support, and itinerary-level and multi-destination representation.

#### 2.4.2 Analysis refinement and synthesis

The coded findings were compared across studies to identify similarities, differences, and gaps in semantic tourism modelling. Particular attention was given to whether the reviewed studies remained focused on entity-level tourism knowledge or whether they represented itinerary-level structures. Overlapping categories were merged and thematic boundaries clarified to produce a coherent analytical structure. Thematic analysis provides a flexible yet systematic method for synthesizing interdisciplinary literature in ontology engineering and tourism informatics (Vaismoradi et al., 2013). The resulting thematic framework forms the basis for findings presented in Section 3. The overall study selection procedure based on PRISMA is illustrated in Figure 2.

### 2.5 Ethics approval

As this study constitutes a systematic literature review of published, publicly accessible academic literature, it does not involve primary data collection from human participants, surveys, interviews, or experiments. Therefore, ethics approval was not required for this research.

## 3 Results

### 3.1 Background of the selected studies

Following the PRISMA-based selection process, 21 studies were included in the final review. The reviewed literature covers ontology and knowledge graph-based approaches in tourism systems, including cultural tourism knowledge graphs, semantic retrieval, tourism recommendation, ontology development, route planning, and decision-support applications.

The selected studies show that semantic technologies are being used in different ways through tourism research. Some studies focus on formal ontology development and semantic modelling, such as Haridy et al. (2023), Pinto et al. (2022), Ojino et al. (2022), Abbasi-Moud et al. (2022), and Fang et al. (2021). Other studies focus on knowledge graph construction, tourism knowledge organization, semantic retrieval, or graph-based recommendation, including Chessa et al. (2023), Fan and Chen (2024), Gao et al. (2024), Jiang and Han (2025), Xu et al. (2025), Zuo and Li (2025), and Cadeddu et al. (2024). Route and recommendation-oriented studies are also present, including Li (2024), Ding (2024), Gao et al. (2023), and Casillo et al. (2025).

Overall, the reviewed studies show that ontology and knowledge graph approaches are useful for organizing tourism knowledge, linking tourism entities, supporting recommendations, and improving semantic retrieval. However, many studies remain focused on entity-level representation, such as attractions, accommodation, cultural resources, tourism services, user preferences, or destination information. Fewer studies explicitly model itinerary-level structures, such as Trip, Itinerary, Segment, Temporal Relation and Route Semantic. This pattern supports the main focus of the review, which is to assess whether existing tourism semantic systems have moved beyond entity-centered modelling toward multi-destination itinerary representation.

### 3.2 Summary of study characteristics

Table 4 summarizes the main characteristics extracted from the 21 included studies. The table reports each study’s approach type, semantic method, tourism domain, application focus, evaluation type, itinerary-level representation.

**Table 4:** Summary of ontology-based tourism studies and their characteristics.

Study	Author (year)	Approach type	Semantic method	Application focus	Evaluation type	Itinerary-level representation
S1	Zhang (2026)	KG construction	Knowledge graph, multimodal model	Cultural tourism integration	System/model evaluation	Not explicit
S2	Wang et al. (2025)	KG construction	Knowledge graph, LLM verification	Tibetan cultural tourism KG	System/model evaluation	Not explicit
S3	Jiang and Han (2025)	Recommendation	Knowledge graph	Attraction recommendation	Recommendation evaluation	Not explicit
S4	Xu et al. (2025)	Recommendation	Location knowledge graph	Tourist attraction recommendation	Recommendation evaluation	Not explicit
S5	Zuo and Li (2025)	Smart tourism service	Knowledge graph, big data	Resource optimization	System evaluation	Partial
S6	Li (2024)	Route planning	KG-based recommendation, RippleNet, GA	Route planning	Route/recommendation evaluation	Partial
S7	Fan and Chen (2024)	KG construction	Ontology + KG (CuPe-KG)	Tourism resources	KG evaluation	Not explicit
S8	Gao et al. (2024)	Decision support	Tourism-oriented KG	Tourist preference and decision support	Decision-support evaluation	Partial
S9	Ding (2024)	Route recommendation	GAT/graph-based method	Attraction and route recommendation	Algorithm evaluation	Partial
S10	Haridy et al. (2023)	Ontology development	Ontology, NLP	Tourism ontology case study	Ontology/methodology evaluation	Not explicit
S11	Chessa et al. (2023)	KG methodology	Knowledge graph + Ontology	Tourism domain KG generation	KG methodology evaluation	Partial
S12	Ojino et al. (2022)	Rule-based reasoning	Ontology, rules	Hotel room personalization	Reasoning/system evaluation	Not explicit
S13	Do et al. (2021)	Question answering	KG, deep learning	Vietnamese tourism QA	QA evaluation	Not explicit
S14	Pinto et al. (2022)	Ontology modelling	Ontology	Urban cultural heritage tourism	Ontology evaluation	Partial
S15	Casillo et al. (2025)	Recommender system	Semantic/multimodal recommender	Cultural tourism	Recommender evaluation	Partial
S16	Aghaei et al. (2022)	Question answering	Knowledge graph	Tourism QA	QA evaluation	Not explicit
S17	Gao et al. (2023)	Explainable recommendation	Knowledge graph	Travel recommendation interpretability	Recommendation evaluation	Not explicit
S18	Fan and Wang (2022)	KG construction	Knowledge graph, GAT	Intangible cultural heritage	KG/extraction evaluation	Not explicit
S19	Abbasi-Moud et al. (2022)	Recommender system	Fuzzy ontology	Context-aware tourism recommendation	Recommendation evaluation	Not explicit
S20	Fang et al. (2021)	Semantic retrieval	Event ontology/semantic retrieval	Cultural tourism events	Retrieval evaluation	Not explicit
S21	Cadeddu et al. (2024)	KG-enhanced application	KG, language models	Tourism accommodation offers	System evaluation	Not explicit

KG, knowledge graph; RDF, resource description framework; OWL, web ontology language; QA, question answering; GAT, graph attention network; GA, genetic algorithm; LLM, large language model. Itinerary-level representation was classified as explicit, partial, or not explicit. Explicit refers to direct modelling of itinerary-related constructs such as trip, itinerary, segment, temporal relation, route, sequence, or transition. Partial refers to route, distance, time, location-linking, recommendation-path, or decision-support elements that are present but not formalized as complete itinerary semantics. Not explicit refers to ontology or knowledge graph use for tourism entities without itinerary-level modelling.

The reviewed studies can be grouped into several broad categories. First, ontology-oriented studies focus on ontology development, semantic modelling, rule-based reasoning, or fuzzy ontology-based recommendation. Second, knowledge graph-oriented studies focus on tourism resource modelling, cultural tourism knowledge graphs, attraction recommendation, semantic retrieval, or question answering. Third, route and recommendation-oriented studies apply semantic or graph-based methods to support tourism recommendation, route planning, or decision support.

The study characteristics indicate that ontology and knowledge graph approaches are widely used to represent tourism knowledge. However, explicit multi-destination itinerary representation remains limited. Most studies support tourism knowledge organisation, recommendation, retrieval, or decision support, but do not fully represent travel sequences as formal semantic structures. Therefore, the following analysis examines whether the reviewed studies include itinerary-level constructs such as Itinerary and Route.

### 3.3 Findings

To analyze the selected studies beyond descriptive comparison, six themes (T1–T6) were developed deductively, based on the research questions, data extraction categories, and coding procedure described in Section 2.4.1. These themes reflect both the methodological orientation of the studies and their application focus in tourism systems.

Theme identification method:

- Repeated coding of study objectives semantic methods, tourism application areas, and evidence of itinerary-level representation;
- Grouping similar methodological features;
- Refining thematic classifications through comparison with the data extraction matrix to improve consistency across the 21 studies.

This coding approach follows deductive thematic analysis as described by Braun and Clarke (2006). Table 5 maps each study to the six thematic areas and reports the number and percentage of studies under each theme.

**Table 5:** Thematic distribution of selected studies across ontology-based tourism research dimensions.

Study	Study (author, year)	T1	T2	T3	T4	T5	T6
S1	Zhang (2026)		✓				
S2	Wang et al. (2025)		✓				
S3	Jiang and Han (2025)		✓	✓			
S4	Xu et al. (2025)		✓	✓			
S5	Zuo and Li (2025)		✓			✓	
S6	Li (2024)		✓	✓		✓	✓
S7	Fan and Chen (2024)		✓				
S8	Gao et al. (2024)		✓			✓	✓
S9	Ding (2024)		✓	✓		✓	✓
S10	Haridy et al. (2023)	✓					
S11	Chessa et al. (2023)	✓	✓				
S12	Ojino et al. (2022)	✓		✓			
S13	Do et al. (2021)		✓		✓		
S14	Pinto et al. (2022)	✓					
S15	Casillo et al. (2025)			✓			
S16	Aghaei et al. (2022)		✓		✓		
S17	Gao et al. (2023)		✓	✓			
S18	Fan and Wang (2022)		✓				
S19	Abbasi-Moud et al. (2022)	✓		✓			
S20	Fang et al. (2021)	✓			✓		
S21	Cadeddu et al. (2024)		✓				

Theme	Description	Number of studies	Percentage
T1	Ontology and semantic modelling	6	28.6%
T2	Knowledge graph construction and enrichment	15	71.4%
T3	Tourism recommendation and personalization	8	38.1%
T4	Semantic retrieval and question answering	3	14.3%
T5	Route planning and decision support	4	19.0%
T6	Itinerary-level and multi-destination representation	3	14.3%

A study may address multiple themes; therefore, percentages do not sum to 100%. ✓ indicates the study addresses the corresponding theme.

The thematic mapping shows stronger concentration in knowledge graph construction and enrichment (T2), and tourism recommendation and personalization (T3). In contrast, route planning and decision-support applications (T5) appear less frequently, while formal itinerary semantic constructs (T6) are the least represented. This pattern supports the review finding that tourism semantic systems are commonly used for data organization, retrieval, and recommendation, but less often for formal representation of itinerary-level structures such as Itinerary and Route.

#### 3.3.1 Ontology and knowledge graph-based approaches in tourism planning

The thematic distribution in Table 5 reveals that ontology and knowledge graph-based approaches are used across different tourism planning and related tourism applications. The studies are not limited to multi-destination planning systems. They include ontology development, tourism knowledge graph construction, semantic retrieval, question answering, recommendation, route planning, and decision-support applications. This wider coverage is relevant because it shows how semantic technologies are currently used in tourism and whether they have progressed toward itinerary-level representation.

At the ontology-centric end, studies such as Haridy et al. (2023) treat ontology construction as the principal outcome, emphasizing methodological transparency through ontology-driven conceptual modelling and NLP-supported term extraction. Pinto et al. (2022) similarly apply modular ontology engineering within cultural heritage, capturing entity hierarchies and functional relationships among tourism objects. Ojino et al. (2022) apply ontology and rule-based reasoning for hotel room personalization. Abbasi-Moud et al. (2022) use a fuzzy ontology-based approach for context-aware tourism recommendation, and Fang et al. (2021) apply semantic retrieval for cultural tourism events. These studies show that ontology-based approaches are useful for organizing tourism concepts and supporting semantic interpretation, but they mainly focus on tourism entities, services, events, or preferences rather than complete itinerary structures.

A COMMON pattern across recent research is the integration of ontologies into scalable knowledge-graph (KG) infrastructures. Fan and Chen (2024), Chessa et al. (2023), Zhang (2026), Wang et al. (2025), Fan and Wang (2022), and Cadeddu et al. (2024) focus on knowledge graph construction or enrichment in tourism-related domains. Other studies apply knowledge graphs for attraction recommendation, semantic retrieval, question answering, or user preference modelling, including Jiang and Han (2025), Xu et al. (2025), Do et al. (2021), Aghaei et al. (2022), and Gao et al. (2023). These studies indicate that knowledge graphs are commonly used to connect tourism entities and support retrieval or recommendation tasks. However, their graph structures are mostly used to organize tourism resources, attractions, cultural content, accommodation, or user preferences.

A smaller group of studies is more closely related to route planning and decision support. Li (2024) combines knowledge graph-based recommendation with route planning, while Ding (2024) applies graph-based methods for tourist attraction and route recommendation. Gao et al. (2024) uses a tourism-oriented knowledge graph for preference mining and decision support, and Zuo and Li (2025) links knowledge graphs with smart tourism services and resource optimization. Zhang (2026) also reports route planning support within a cultural tourism knowledge graph context. These studies move closer to travel planning, but the itinerary logic is often handled through recommendation or algorithmic processes rather than through explicit semantic modelling of Itinerary or Route constructs.

#### 3.3.2 Semantic design and evaluation practices

The reviewed studies show variation in how ontology and knowledge graph-based tourism systems are designed and evaluated. Ontology-oriented studies tend to describe semantic structures more explicitly. Haridy et al. (2023), Pinto et al. (2022), and Ojino et al. (2022) report concepts, relationships, rules, or class-based structures that support tourism knowledge modelling. Fang et al. (2021) and Abbasi-Moud et al. (2022) also apply semantic modelling, but within more specific application contexts such as cultural tourism event retrieval and context-aware recommendation.

Knowledge graph-based studies generally place stronger emphasis on entity connection, data enrichment, and application use. Fan and Chen (2024), Chessa et al. (2023), Zhang (2026), Wang et al. (2025), Fan and Wang (2022), and Cadeddu et al. (2024) focus on constructing or enriching tourism-related knowledge graphs. Other studies apply knowledge graphs to recommendation, question answering, user preference modelling, and decision support (Jiang and Han, 2025; Xu et al., 2025; Do et al., 2021; Aghaei et al., 2022; Gao et al., 2023; Gao et al., 2024). These studies usually describe tourism entities and relationships, but the level of detail on semantic constraints, reasoning mechanisms, or itinerary-related structures differs across studies.

Evaluation practices also vary. Ontology-oriented studies commonly report validation through modelling checks, reasoning, expert review, or methodology-based evaluation (Haridy et al., 2023; Pinto et al., 2022). Recommendation and route-related studies often use system-level metrics such as recommendation performance, ranking accuracy, route quality, or algorithm comparison (Li, 2024; Ding, 2024; Jiang and Han, 2025). Knowledge graph and semantic retrieval studies may evaluate graph construction, retrieval accuracy, question answering performance, or usefulness of linked tourism data (Chessa et al., 2023; Fan and Chen, 2024; Aghaei et al., 2022).

Overall, the reviewed studies show that semantic design and evaluation practices are not reported consistently. Formal ontology studies tend to explain classes, relationships, and reasoning more clearly. Knowledge graph and application-oriented studies tend to focus more on data integration, retrieval, recommendation, or system performance. This makes cross-study comparison difficult and supports the need for clearer reporting of classes, entities, relationships, semantic constraints, reasoning mechanisms, validation methods, and itinerary-level constructs in future tourism semantic systems.

#### 3.3.3 Representation of multi-destination tourism

Thematic analysis indicates limited progress in explicitly modelling multi-destination or itinerary-based constructs. Most studies model tourism entities such as attractions, accommodation, cultural resources, events, services, user preferences, or destination information. These models are useful for knowledge organization, retrieval, recommendation, and decision support, but they do not usually represent travel sequences as formal semantic structures.

Ontology-oriented studies such as Pinto et al. (2022), Ojino et al. (2022), Haridy et al. (2023), Abbasi-Moud et al. (2022), and Fang et al. (2021) structure tourism knowledge through concepts, relationships, rules, or semantic retrieval mechanisms. However, these studies mainly focus on tourism resources, events, services, or user context. They do not explicitly define itinerary-level constructs such as Trip, Itinerary, Segment, Temporal Relation, Route Semantic, Sequence, or Transition. To provide a more detailed evidence trail, the retained studies were further assessed against five itinerary-level semantic constructs: Trip Class, Itinerary Class, Segment Class, Temporal Relation, and Route Semantic. These constructs were used to determine whether each study moved beyond general tourism entity modelling toward explicit representation of travel sequence, transition, time, and route dependency. The assessment is presented in Table 6.

**Table 6:** Assessment of itinerary-level semantic constructs in the retained studies.

Study	Trip class	Itinerary class	Segment class	Temporal relation	Route semantic	Interpretation
S1 Zhang (2026)	No	No	No	Partial	Partial	Supports route-related cultural tourism KG, but does not formalize itinerary classes
S2 Wang et al. (2025)	No	No	No	No	No	Cultural tourism KG construction, no itinerary-level constructs
S3 Jiang and Han (2025)	No	No	No	No	Partial	Attraction recommendation using KG, but itinerary structure is not explicit
S4 Xu et al. (2025)	No	No	No	No	Partial	Attraction recommendation using location KG, no formal itinerary classes
S5 Zuo and Li (2025)	No	No	No	Partial	Partial	Smart tourism and resource optimization with partial route/resource relation
S6 Li (2024)	No	Partial	No	Partial	Partial	Route planning is present, but itinerary constructs are mainly algorithmic
S7 Fan and Chen (2024)	No	No	No	No	No	Tourism resource KG, no itinerary-level modelling
S8 Gao et al. (2024)	No	Partial	No	Partial	Partial	Decision support and spatiotemporal KG elements, but incomplete itinerary semantics
S9 Ding (2024)	No	Partial	No	Partial	Partial	Route recommendation present, but not formal itinerary ontology
S10 Haridy et al. (2023)	No	No	No	No	No	Ontology development case study, no itinerary-level constructs
S11 Chessa et al. (2023)	No	No	No	No	Partial	Tourism KG methodology, limited route/destination relationship representation
S12 Ojino et al. (2022)	No	No	No	No	No	Hotel personalization ontology, no itinerary-level constructs
S13 Do et al. (2021)	No	No	No	No	No	Tourism QA over KG, no itinerary-level modelling
S14 Pinto et al. (2022)	No	No	No	No	Partial	Urban cultural heritage ontology, limited spatial relation but no itinerary structure
S15 Casillo et al. (2025)	No	Partial	No	Partial	Partial	Recommender system supports cultural tourism experience, but itinerary classes are not formalized
S16 Aghaei et al. (2022)	No	No	No	No	No	Tourism QA over KG, no itinerary-level modelling
S17 Gao et al. (2023)	No	No	No	No	Partial	Explainable travel recommendation using KG, no formal itinerary constructs
S18 Fan and Wang (2022)	No	No	No	No	No	Heritage KG construction, no itinerary-level modelling
S19 Abbasi-Moud et al. (2022)	No	No	No	No	No	Context-aware fuzzy ontology recommendation, no itinerary structure
S20 Fang et al. (2021)	No	No	No	Partial	No	Event semantic retrieval, limited temporal aspect but no itinerary structure
S21 Cadeddu et al. (2024)	No	No	No	No	No	Accommodation KG application, no itinerary-level modelling

“Yes” indicates that the construct is explicitly represented as a semantic class or relationship in the ontology, knowledge graph, or semantic model. “Partial” indicates that the study includes route, location, time, distance, recommendation path, or decision-support elements, but does not formalize the construct as a complete itinerary-level semantic class or relationship. “No” indicates that the construct is not explicitly represented in the ontology, knowledge graph, or semantic model.

Knowledge graph studies also show a similar pattern. Fan and Chen (2024), Chessa et al. (2023), Wang et al. (2025), Fan and Wang (2022), Cadeddu et al. (2024), Do et al. (2021), Aghaei et al. (2022), and Gao et al. (2023) use knowledge graphs to connect tourism entities, support retrieval, organise cultural tourism content, or improve recommendation and question answering. These studies improve the connectivity of tourism data, but most do not treat itineraries as semantic objects within the graph.

A smaller group of studies partially addresses route planning or decision support. Li (2024) includes route planning through a knowledge graph-based recommendation model, while Ding (2024) applies graph-based methods for attraction and route recommendation. Gao et al. (2024) uses a tourism-oriented knowledge graph for preference mining and decision support, and Zhang (2026) reports route planning support within a cultural tourism knowledge graph context. Zuo and Li (2025) also connects knowledge graphs with smart tourism services and resource optimisation. These studies are closer to travel planning, but the route or itinerary component is usually handled through recommendation, optimisation, or decision-support logic rather than through formal itinerary semantic constructs.

This distinction is important. A study may support route recommendation or destination selection without formally modelling an itinerary. Based on the itinerary-level assessment, most reviewed studies were classified as either “Not explicit” or “Partial” in their representation of Itinerary and Route constructs. This supports the finding that ontology and knowledge graph-based tourism systems are still largely entity-centred, while formal multi-destination itinerary semantics remain underdeveloped.

#### 3.3.4 Research gaps identified

The synthesis across Sections 3.3.1 to 3.3.3 identifies three dominant research gaps and clarifies the underlying factors contributing to them.

##### 3.3.4.1 Limited itinerary-level formalization

Few studies represent trip structures or travel sequences as formal semantic entities. Route planning and recommendation studies such as Li (2024), Ding (2024), Gao et al. (2024), Zhang (2026), and Zuo and Li (2025) move closer to travel planning, but their itinerary logic is mostly expressed through algorithmic or application-level processes. Formal constructs such as Trip Class, Itinerary Class, Segment Class, Temporal Relation, and Route Semantic are still rarely defined as part of the ontology or knowledge graph structure.

##### 3.3.4.2 Fragmented validation and low interoperability

The reviewed studies use different evaluation approaches. Ontology-oriented studies tend to report modelling checks, reasoning, expert review, or methodology-based validation (Haridy et al., 2023; Pinto et al., 2022). Recommendation, retrieval, question answering, and route-related studies often use system-level metrics such as ranking performance, recommendation accuracy, retrieval performance, question answering accuracy, or route quality (Li, 2024; Ding, 2024; Jiang and Han, 2025; Aghaei et al., 2022). These differences make direct comparison difficult, especially when ontology-level validity and system-level performance are evaluated using different criteria.

##### 3.3.4.3 Insufficient integration of dynamic data and reasoning

Only a small number of studies address route, distance, time, transport, or decision-support elements. Even when such elements are present, they are not always represented as semantic constraints that can support reasoning over travel sequence, duration, transition, or inter-destination dependency. This limits the ability of tourism systems to explain how destinations are connected within a multi-destination itinerary.

Overall, the reviewed studies show strong use of ontology and knowledge graph approaches for tourism knowledge organization, recommendation, retrieval, and decision support. However, the formal representation of itinerary-level semantics remains limited. Future tourism ontology and knowledge graph models should therefore give more attention to explicit itinerary-level constructs so that multi-destination travel can be represented and queried more clearly.

## 4 Discussion

### 4.1 Development of ontology and knowledge graph-based tourism systems

The findings indicate that ontology and knowledge graph-based tourism research has developed across several directions, including ontology development, knowledge graph construction, semantic retrieval, recommendation, route planning, and decision-support applications. Ontology-oriented studies such as Pinto et al. (2022), Ojino et al. (2022), Haridy et al. (2023), Abbasi-Moud et al. (2022), and Fang et al. (2021) show how semantic structures can be used to organize tourism concepts, relationships, rules, events, services, and user preferences.

The reviewed studies also show increased use of knowledge graph-based approaches. Fan and Chen (2024), Chessa et al. (2023), Zhang (2026), Wang et al. (2025), Fan and Wang (2022), and Cadeddu et al. (2024) focus on knowledge graph construction or enrichment in tourism-related domains. Other studies apply knowledge graphs for attraction recommendation, question answering, user preference modelling, and travel recommendation, including Jiang and Han (2025), Xu et al. (2025), Do et al. (2021), Aghaei et al. (2022), and Gao et al. (2023). These studies suggest that knowledge graphs are useful for connecting tourism entities and supporting applied tourism services.

Travel planning inherently involves interconnected locations, travel transitions, and temporal constraints, all of which naturally align with graph structures (Lew and McKercher, 2006). Ontologies contribute semantic clarity and conceptual structure, while knowledge graphs provide scalability and operational flexibility (Feilmayr and Wöß, 2016). However, Tables 4, 6 show that itinerary-level representation remains limited. Several studies support route planning or decision support, such as Li (2024), Ding (2024), Gao et al. (2024), Zuo and Li (2025), and Zhang (2026). Even so, most of these studies handle itinerary logic through recommendation, optimization, or application-level processes rather than explicit semantic constructs.

This suggests that the development of tourism semantic systems has been stronger in knowledge organization, recommendation, retrieval, and data integration than in formal itinerary-level modelling. Future work would benefit from combining ontology-level conceptual clarity with knowledge graph scalability so that multi-destination travel structures can be represented more clearly within tourism planning systems (Poveda-Villalón et al., 2022).

### 4.2 Semantic design and evaluation practices in tourism

The reviewed studies reveal several recurring ontology design patterns. Most tourism ontologies begin with hierarchical structures representing core entities such as destinations, points of interest, accommodation, transport and services. These concepts are linked through object properties describing spatial, functional, or contextual relationships, including location, proximity, service provision, or thematic categorization. Knowledge graph-based studies represent similar tourism entities as connected nodes and relationships, often to support data integration, retrieval, recommendation, or decision support.

The level of modelling detail varies across the 21 studies. Ontology-oriented studies generally provide clearer explanation of concepts, relationships, rules, and validation steps. Knowledge graph and application-oriented studies, on the other hand, tend to give more attention to entity linking, data enrichment, recommendation performance, retrieval accuracy, or decision-support output. This difference matters because strong system performance does not always mean that the underlying semantic structure is complete, reusable, or suitable for itinerary-level reasoning.

The reviewed studies also differ in how they evaluate their systems. Ontology-oriented studies tend to report modelling checks, reasoning, expert review, rule evaluation, or methodology-based validation. Recommendation and route-related studies often use system-level evaluation, such as recommendation accuracy, ranking performance, route quality, or algorithm comparison. Knowledge graph, semantic retrieval, and question answering studies may evaluate graph construction, retrieval accuracy, question answering performance, or usefulness of linked tourism data. These different evaluation practices make cross-study comparison difficult because ontology-level validity and system-level performance are not always assessed using the same criteria.

This issue is particularly relevant for multi-destination tourism planning. Itinerary modelling requires more than the representation of tourism entities. It also requires semantic representation of sequence ordering, travel duration, spatial transition, temporal dependency, and route relationships. Ontology engineering guidelines emphasise structured validation procedures, including competency questions, reasoning verification, and iterative refinement (Poveda-Villalón et al., 2022). Based on the reviewed studies, such practices are still not reported consistently in tourism ontology and knowledge graph research. Future studies would benefit from clearer reporting of classes, entities, relationships, semantic constraints, reasoning mechanisms, evaluation methods, and itinerary-level constructs.

### 4.3 Limitations in multi-destination tourism representation

The reviewed studies show limited semantic representation of multi-destination tourism planning. As shown in Tables 4, 6, many studies model tourism entities such as attractions, cultural resources, accommodation, events, services, user preferences, or destination information. These models support knowledge organisation, retrieval, recommendation, question answering, and decision support, but they do not usually represent trip-level itinerary structures as formal semantic constructs.

Several studies move closer to travel planning. Li (2024) includes route planning through a knowledge graph-based recommendation model, while Ding (2024) applies graph-based methods for attraction and route recommendation. Gao et al. (2024) uses a tourism-oriented knowledge graph for preference mining and decision support, and Zhang (2026) reports route planning support within a cultural tourism knowledge graph context. Zuo and Li (2025) also links knowledge graphs with smart tourism services and resource optimisation. However, in these studies, route or itinerary logic is mainly handled through recommendation, optimisation, or decision-support processes rather than through explicit semantic constructs such as Trip, Itinerary, Segment, Temporal Relation, Route Semantic, Sequence, or Transition.

This creates a modelling limitation for multi-destination tourism planning. When itinerary semantics are not represented within the ontology or knowledge graph structure, the system may still recommend routes or destinations, but the reasoning behind sequence, travel transition, time, distance, and dependency may be less explicit. This affects explainability and reuse because the itinerary is treated more as an output of an algorithm than as a structured semantic object.

This issue is also linked to the broader design pattern observed in the review. Ontology and knowledge graph-based tourism systems are effective for linking tourism entities and supporting recommendation or retrieval. However, itinerary-level planning requires additional modelling of temporal and spatial dependencies. By representing trips, destinations, segments, and travel transitions semantically, future systems could provide clearer explanations of how travel sequences are formed and how planning constraints influence itinerary generation.

### 4.4 Implications for future tourism ontology and knowledge graph development

The findings suggest that future tourism ontology and knowledge graph development should give more attention to itinerary-level representation. Rather than treating itinerary generation only as an output of recommendation or optimisation algorithms, future models could represent itinerary structures as semantic entities. This includes concepts such as Trip, Itinerary, Stop, Segment, Sequence, Transition, Temporal Relation, and Route Semantic. These constructs can help describe how destinations are connected, how travel order is formed, and how time, distance, and transport constraints influence a travel plan.

A second implication concerns validation. The reviewed studies show that ontology-oriented research and application-oriented systems often use different evaluation approaches. Future studies should report ontology or knowledge graph validation more clearly, including competency questions, reasoning checks, expert review, schema validation, or other procedures that explain how the semantic structure was tested. This would make it easier to compare studies and assess whether the model supports itinerary-level reasoning, rather than only application-level performance.

A third implication is the need to combine ontology-level meaning with knowledge graph scalability. Knowledge graphs are useful for connecting large tourism datasets and supporting recommendation, retrieval, and decision support. However, the semantic layer should still describe the meaning of key tourism concepts, relationships, constraints, and itinerary structures. A hybrid approach could allow tourism systems to benefit from graph-based data integration while retaining clearer semantic representation of travel sequences and inter-destination dependencies.

For practice, technology developers and destination managers may use these findings as a guide when designing tourism systems. Instead of modelling only points of interest, systems should also consider trip structure, route relationships, time constraints, transport links, and explanation of recommendation logic. This can support more transparent and interoperable tourism planning systems, especially in environments where tourism data are spread across multiple platforms and stakeholders.

## 5 Conclusion

This study conducted a systematic literature review to examine ontology and knowledge graph-based approaches in tourism planning, particularly their capability to support itinerary-level representation. Guided by the PRISMA framework (Page et al., 2021), 21 studies were identified, screened and analyzed from major academic databases, providing a structured overview of how ontology and knowledge graph-based approaches have been applied in tourism knowledge modelling and intelligent tourism systems.

The findings show that ontology and knowledge graph-based approaches are widely used to organize tourism knowledge, connect tourism entities, support semantic retrieval, and improve recommendation systems. Most studies focus on modelling tourism entities such as destinations, attractions and services using ontologies and knowledge graphs (Feilmayr and Wöß, 2016). Several studies integrate ontologies with intelligent tourism systems to enhance personalized recommendations within smart tourism environments (Gretzel et al., 2015). However, many tourism ontologies remain destination-centric, with limited explicit representation of multi-destination travel structures such as itinerary relationships, destination sequences and temporal constraints. Given that modern travel behavior increasingly involves multi-stop and interconnected patterns (Lew and McKercher, 2006), this absence represents an important research gap.

This review contributes by organizing recent ontology and knowledge graph-based tourism studies according to their semantic approach, application focus, evaluation method, and level of itinerary representation. The synthesis highlights the need for clearer modelling of travel sequences, inter-destination relationships, temporal constraints, and route dependencies. It also highlights the need for stronger validation practices, including competency-question testing, reasoning verification, expert review, and clearer reporting of semantic structures.

Several limitations should be acknowledged. The review was restricted to selected databases and English-language publications, which may have excluded relevant studies elsewhere. Additionally, the diversity of research designs limited the possibility of quantitative synthesis such as meta-analysis. Future research should explore ontology and knowledge graph-based tourism planning models that explicitly represent multi-destination travel structures and validate them within real-world contexts.

## Data Availability

The original contributions presented in the study are included in the article/supplementary material, further inquiries can be directed to the corresponding authors.

## References

[ref1] Abbasi-Moud Z. Hosseinabadi S. Kelarestaghi M. Eshghi F. (2022). CAFOB: context-aware fuzzy-ontology-based tourism recommendation system. Expert Syst. Appl. 199:116877. doi: 10.1016/j.eswa.2022.116877

[ref2] Aghaei S. Raad E. Fensel A. (2022). Question answering over knowledge graphs: a case study in tourism. IEEE Access 10, 69788–69801. doi: 10.1109/ACCESS.2022.3187178

[ref3] Alexander P. A. (2020). Methodological guidance for systematic reviews in emerging research domains. Rev. Educ. Res. 90, 6–23. doi: 10.3102/0034654319854352, 38293548

[ref4] Braun V. Clarke V. (2006). Using thematic analysis in psychology. Qual. Res. Psychol. 3, 77–101. doi: 10.1191/1478088706qp063oa

[ref5] Cadeddu A. Chessa A. De Leo V. Fenu G. Motta E. Osborne F. . (2024). Optimizing tourism accommodation offers by integrating language models and knowledge graph technologies. Information 15:398. doi: 10.3390/info15070398

[ref6] Casillo M. Colace F. Lorusso A. Santaniello D. Valentino C. (2025). Integrating physical and virtual experiences in cultural tourism: an adaptive multimodal recommender system. IEEE Access 13, 28353–28368. doi: 10.1109/ACCESS.2025.3539205

[ref7] Chessa A. Fenu G. Motta E. Osborne F. Reforgiato Recupero D. Salatino A. . (2023). Data-driven methodology for knowledge graph generation within the tourism domain. IEEE Access 11, 67567–67599. doi: 10.1109/ACCESS.2023.3292153

[ref9] Ding T. (2024). Multi-source auxiliary information tourist attraction and route recommendation algorithm based on graph attention network. J. Intell. Syst. 33:20240070. doi: 10.1515/jisys-2024-0070

[ref10] Do P. Phan T. H. V. Gupta B. B. (2021). Developing a Vietnamese tourism question answering system using knowledge graph and deep learning. ACM Trans. Asian Low-Resour. Lang. Inf. Process. 20, 1–18. doi: 10.1145/3453651

[ref11] Fan Z. Chen C. (2024). CuPe-KG: cultural perspective–based knowledge graph construction of tourism resources via pretrained language models. Inf. Process. Manag. 61:103646. doi: 10.1016/j.ipm.2024.103646

[ref12] Fan T. Wang H. (2022). Research of Chinese intangible cultural heritage knowledge graph construction and attribute value extraction with graph attention network. Inf. Process. Manag. 59:102753. doi: 10.1016/j.ipm.2021.102753

[ref13] Fang H. Chen C. Wu X. Ye X. (2021). FESRCT: a framework for the event semantic retrieval of cultural tourism. J. Comput. Cult. Herit. 14, 1–23. doi: 10.1145/3451994

[ref14] Feilmayr C. Wöß W. (2016). An analysis of ontologies and their success factors for application to business. Data Knowl. Eng. 101, 1–23. doi: 10.1016/j.datak.2015.11.003

[ref15] Gao J. Peng P. Lu F. Claramunt C. Qiu P. Xu Y. (2024). Mining tourist preferences and decision support via tourism-oriented knowledge graph. Inf. Process. Manag. 61:103523. doi: 10.1016/j.ipm.2023.103523

[ref16] Gao J. Peng P. Lu F. Claramunt C. Xu Y. (2023). Towards travel recommendation interpretability: disentangling tourist decision-making process via knowledge graph. Inf. Process. Manag. 60:103369. doi: 10.1016/j.ipm.2023.103369

[ref17] Gómez-Pérez A. (1999). Ontological engineering: a state of the art. Knowl. Eng. Rev. 14, 3–32. doi: 10.1017/S0269888900000341

[ref18] Gretzel U. Sigala M. Xiang Z. Koo C. (2015). Smart tourism: foundations and developments. Electron. Mark. 25, 179–188. doi: 10.1007/s12525-015-0196-8

[ref9010] Gruber T. R. (1993). A translation approach to portable ontology specifications. Knowledge Acquisition, 5, 199–220. doi: 10.1006/knac.1993.1008

[ref19] Haridy S. Ismail R. M. Badr N. Hashem M. (2023). An ontology development methodology based on ontology-driven conceptual modeling and natural language processing: tourism case study. Big Data Cogn. Comput. 7:101. doi: 10.3390/bdcc7020101

[ref20] Izani U. M. Che Lah N. S. (2025). Ontology driven platform for tourism insights in Malaysia. Math. Sci. Inform. J. 6, 193–202. doi: 10.24191/mij.v6i2.9044

[ref21] Jiang Q. Han Y. (2025). Knowledge graph-driven personalized attractions recommendations with tourists' long- and short-term interest modeling. Expert Syst. Appl. 275:127094. doi: 10.1016/j.eswa.2025.127094

[ref9001] Joanna Briggs Institute. (2020). JBI Manual for Evidence Synthesis. JBI. doi: 10.46658/JBIMES-20-01.

[ref22] Kitchenham B. Charters S. (2007). Guidelines for Performing systematic Literature Reviews in Software Engineering. Keele, UK: Keele University.

[ref9002] Lew A. McKercher B. (2006). Modeling tourist movements: a local destination analysis. Ann. Tour. Res., 33, 403–423. doi: 10.1016/j.annals.2005.12.002

[ref23] Li Y. (2024). Digital tourism recommendation and route planning model design based on RippleNet and improved GA. Informatica 48, 133–152. doi: 10.31449/inf.v48i10.5790

[ref9003] Mallett R. Hagen-Zanker J. Slater R. Duvendack M. (2012). The benefits and challenges of using systematic reviews in international development research. J. Dev. Eff. 4, 445–455. doi: 10.1080/19439342.2012.711342

[ref9004] Moher D. Liberati A. Tetzlaff J. Altman D. G. The PRISMA Group. (2009). Preferred reporting items for systematic reviews and meta-analyses: the PRISMA statement. PLOS Medicine. 6:e1000097. doi: 10.1371/journal.pmed.1000097 21603045 PMC3090117

[ref24] Noy N. F. McGuinness D. L. (2001). Ontology Development 101: A guide to Creating your first Ontology. Stanford, CA, USA: Stanford Knowledge Systems Laboratory.

[ref25] Ojino R. Mich L. Mvungi N. (2022). Hotel room personalization via ontology and rule-based reasoning. Int. J. Web Inf. Syst. 18, 369–387. doi: 10.1108/IJWIS-02-2022-0045

[ref9005] Page M. J. McKenzie J. E. Bossuyt P. M. Boutron I. Hoffmann T. C. Mulrow C. D. . (2021). The PRISMA 2020 statement: an updated guideline for reporting systematic reviews. BMJ, 372, n71. doi: 10.1136/bmj.n71 33782057 PMC8005924

[ref9006] Petrosino A. Boruch R. F. Soydan H. Duggan L. Sanchez-Meca J. (2001). Meeting the challenges of evidence-based policy: The Campbell Collaboration. The Ann. Am. Acad. Polit. Soc. Sci., 578, 14–34. doi: 10.1177/000271620157800102

[ref27] Pinto A. Cardinale Y. Dongo I. Ticona-Herrera R. (2022). An ontology for modeling cultural heritage knowledge in urban tourism. IEEE Access 10, 61820–61842. doi: 10.1109/ACCESS.2022.3179664

[ref28] Poveda-Villalón M. Fernández-Izquierdo A. Fernández-López M. García-Castro R. (2022). LOT: an industrial oriented ontology engineering framework. Eng. Appl. Artif. Intell. 111:104755. doi: 10.1016/j.engappai.2022.104755

[ref9007] Sierra-Correa P. C. Cantera Kintz J. R. (2015). Ecosystem-based adaptation for improving coastal planning for sea-level rise: a systematic review for mangrove coasts. Marine Policy, 51, 385–393. doi: 10.1016/j.marpol.2014.09.013

[ref29] Torraco R. J. (2016). Writing integrative literature reviews: guidelines and examples. Hum. Resour. Dev. Rev. 15, 456–476. doi: 10.1177/153448431667160

[ref30] Uschold M. Gruninger M. (1996). Ontologies: principles, methods and applications. Knowl. Eng. Rev. 11, 93–136. doi: 10.1017/S0269888900007793

[ref9008] Vaismoradi M. Turunen H. Bondas T. (2013). Content analysis and thematic analysis: implications for conducting a qualitative descriptive study. Nursing \u0026amp; Health Sciences, 15, 398–405. doi: 10.1111/nhs.12048 23480423

[ref31] Wang K. Yan S. Liu Z. Yuan X. Li F. Jiang B. . (2025). Knowledge-driven 3D content generation: a rule+LLM-verify-based method for constructing a Tibetan cultural and tourism knowledge graph. Electronics 14:4138. doi: 10.3390/electronics14214138

[ref9009] Whittemore R. Knafl K. (2005). The integrative review: updated methodology. J. Adv. Nurs. 52, 546–553. doi: 10.1111/j.1365-2648.2005.03621.x 16268861

[ref32] Xu J. Bai Z. Ma Q. (2025). Tourist attractions recommendation by using enhanced location knowledge graph. IEEE Access 13, 168463–168477. doi: 10.1109/ACCESS.2025.3613788

[ref33] Zhang X. (2026). Construction of cultural tourism integration knowledge graph method based on multimodal large models. J. Appl. Sci. Eng. 30. doi: 10.6180/jase.202607_30.021

[ref34] Zuo J. Li J. (2025). Smart tourism services and resource optimisation based on big data and knowledge graphs. Int. J. Inf. Commun. Technol. 26, 58–74. doi: 10.1504/IJICT.2025.150406, 35009967

